# Simulation study of gas sensor using periodic phononic crystal tubes to detect hazardous greenhouse gases

**DOI:** 10.1038/s41598-022-26079-0

**Published:** 2022-12-13

**Authors:** Zaky A. Zaky, Sagr Alamri, Ensjam I. Zohny, Arafa H. Aly

**Affiliations:** 1 TH-PPM Group, Physics Department, Faculty of Science, Beni-Suef University, Beni-Suef, 62521 Egypt; 2 Department of Mechanical Engineering, College of Engineering, King Khalid University, 61421 Abha, Saudi Arabia

**Keywords:** Fluid dynamics, Acoustics

## Abstract

Here, we investigate a gas sensor model based on phononic crystals of alternating tubes using the transfer matrix method to detect hazardous greenhouse gases. The effect of the thicknesses and cross-sections of all tubes on the performance of the proposed sensor is studied. The results show that longitudinal acoustic speed is a pivotal parameter rather than the mass density variations of the gas samples on the position of the resonant peaks due to its significant impact on the propagation of the acoustic wave. The suggested sensor can be considered very simple and low-cost because it does not need a complicated process to deposit multilayers of different mechanical properties’ materials.

## Introduction

In recent years, there has been a lot of interest in using periodic media in different acoustics^1,2^ and optics applications^3–12^. Acoustic devices with good performance are vital in biosensing^13^, acoustic communication^14^, gas detection^15^, etc. The occurrence of sound wave dispersion and forbidden frequency bands in phononic crystals (PnCs) has increased interest in them. Photonic crystals’ (PCs) acoustical counterparts are PnCs^4,16,17^. PnC is a periodic geometry developed from the expression of lattice vibration, which refers to phonon. The tubes of PnC are structured in a regular pattern, which allows for spatial manipulation of the acoustic characteristics and promotes the reflection of elastic and acoustic waves, resulting in band gaps. Phononic bandgap (PnBG) is proposed to prevent the propagation of a certain frequency range of elastic and acoustic waves within PnC.

PnBG property offers a novel method of regulating vibration and noise^18^. Besides, PnBG can be created using a periodic branched resonator^19^. The ratio of acoustic impedances between successive layers, periodicity, physical properties, and the geometry of the materials will determine the depth, location, and bandwidth of the created PnBG. Kushwaha et al.^20^ investigated the PnCs band structure and studied the acoustic wave control concept. PnCs containing a defect layer have gotten much attention due to the appearance of a very thin defect mode with high transmittance. As a result of the defect mode, the acoustic wave at specific energy can travel through the PnBG. Within the defect layer, the acoustic energy is trapped in the form of a passband, like what’s happening for photons in the defected PC^21^. The defect mode's frequency depends on the defect layer's geometry, position, and nature. The appearance of this resonant mode gives the PnC a chance to be used in different applications such as filters and detectors.

There have been numerous gas detectors developed based on PC and PnCs^22,23^. PnCs are well-known for gas and liquid monitoring because elastic characteristics of materials vary dramatically from other properties such as density, resulting in high performance^24^. Infrared radiation is absorbed and reradiated by hazardous greenhouse gases such as CH_4_CO_2_, N_2_O, and CO_2_ generated from the Earth and trap it. As a result, these gases are considered particularly harmful chemicals^25^. In 2022, Imanian et al.^24^ presented a gas sensor to detect different types of gases such as CH_4_, O_2_, NH_3_, CO_2_, N_2_, Ar, CO_2_, and C_3_H_8_ at different concentrations in the air with a sensitivity of 0.69 MHz kg^–1^ m^3^. Zaki and Basyooni^25^ proposed CO_2_, N_2_O, and CH_4_ gas sensor using the concept of Fano resonance within PnC. Kaya et al.^26^ introduced an acoustic ring resonator for spoof surface acoustic waves (SSAW) on 1D-PnC. Authors observed high *Q-factor* peaks with very small bandwidths that are exceptionally qualified for gas sensing applications. Cicek et al.^27^ numerically and experimentally proposed a gas detector based on SSAWs using 2D-PnC spherical Helmholtz 2D-PnC resonators to detect CO_2_ and CH_4_ in air.

The proposed gas sensor is based on alternating tubes as a one-dimensional PnC (1D-PnC). This study aims to analyze the acoustic interaction with an expansion chamber as a gas sensor using the transfer matrix method (TMM) to detect hazardous greenhouse gases. The frequency response of the defect mode that depends on the change in the gas sample's acoustic properties, which fills the structure, will be studied. The suggested sensor is simple and low-cost because it does not need a complicated process to deposit multilayers of different acoustic properties’ materials. Besides, the proposed sensor does not need a recovery time. It is simple alternating tubes with different cross-sections in periodic sequences. Also, The suggested structure recorded very high linearity between the change in the resonant peak and the acoustic velocity of the sample.

## Basic equations and model design

**Figure 1:**
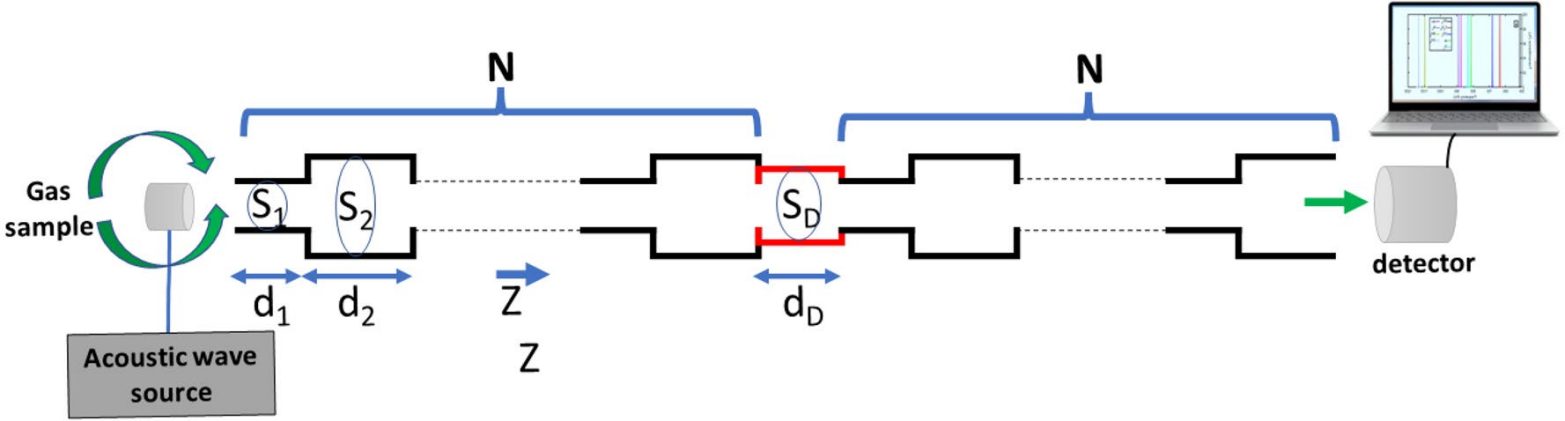
The schematic of the suggested PnC gas sensor with tube sequence of *(M*_*1*_* M*_*2*_*)*^*N*^* M*_*D*_* ( M*_*1*_* M*_*2*_*)*^*N*^.

The intended detector design is depicted in Fig. 1. The unit cell consists of two cavity muffler tubes^28^ with different thicknesses and cross-sections. This unit cell is repeated for *N* finite periods to make a PnC crystal. The first tube has a thickness of *d*_*1*_ and a cross-section of *S*_*1*_. Also, the second tube has a thickness of d_2_ and a cross-section of S_2_. The defect tube has a thickness of d_D_ and a cross-section of S_D_. The cross-sectional areas of resonators are designed perpendicularly to the main tube. The gas sample will fill the proposed sensor. The gas samples are studied at standard pressure (1 atm) and room temperature (20 °C). For a symmetrical periodic structure with a defect, there may be two resonance peaks (topological edge state peak and defect mode peak). However, we need only one peak to prevent the overlap between peaks. So, we preferred to use an asymmetrical structure.

The Helmholtz equation investigates the propagation of the acoustic wave within the proposed sensor structure as^2^:

1 $$\nabla .\left(\frac{1}{\rho }\nabla P\right)+\frac{{\omega }^{2}P}{\rho {c}_{L}^{2}}=0$$

where ${c}_{L}$ is the longitudinal speed of the acoustic wave through the tub, and *P* is the acoustic pressure. TMM is a standard method for studying the transmission and reflection of the incident acoustic and optic waves with multitube structures^29–34^. The following matrix describes the interplay between the proposed system and acoustic waves.

2 $$\left|\begin{array}{ll}{A}_{11}& {A}_{12}\\ {A}_{21}& {A}_{22}\end{array}\right|={\left({M}_{1}{M}_{2}\right)}^{N}{ M}_{D}{ \left({M}_{1}{M}_{2}\right)}^{N},$$

3 $${M}_{i}=\left[\begin{array}{ll}cos(k{d}_{i})& \left(-\frac{i}{{\varnothing }_{i}}\right)sin(k{d}_{i})\\ -i{\varnothing }_{i}sin(k{d}_{i})& cos(k{d}_{i})\end{array}\right],\quad i=\mathrm{1,2},N,$$

where $\mathrm{k}$ is the wave vector ($k=\omega /c)$,${\varnothing }_{i}$ is the reciprocal of the impedance (${Z}_{i}$):

4 $${Z}_{i}=\rho c/{\mathrm{S}}_{\mathrm{i}}.$$

Chebyshev’s polynomial is used to repeat the unit cell $\left({M}_{1}{M}_{2}\right)$ for *N* times. The transmittance of acoustic waves through the proposed hazardous gases sensor can be calculated as a function of transmission:

5 $$t=\frac{2{\mathrm{\varnothing }}_{1}}{\left({A}_{11}+{A}_{12}{\mathrm{\varnothing }}_{1}\right){\mathrm{\varnothing }}_{1}+\left({A}_{21}+{A}_{22}{\mathrm{\varnothing }}_{1}\right)}$$

6 $$T\left(\%\right)=100*{\left|t\right|}^{2}$$

## Results and discussion

### Pure gases calculations

This section studies the acoustic wave propagation within the proposed hazardous gases sensor structure without and with the defect tube. The initial geometrical conditions of the design will be *d*_*1*_ = 0.1 m, *d*_*2*_ = 0.2 m, *d*_*D*_ = 0.15 m, *N* = 6, *S*_*1*_ = 0.1 m^2^, *S*_*2*_ = 0.2 m^2^, *S*_*D*_ = 0.15 m^2^, the acoustic wave will fall on the structure with incident angle ${\theta }_{0}$= 0°, at atmospheric pressure, at the sea level altitude, and the room temperature. Besides, the gas samples inside tubes are stationary. Table 1 clear the mass density and acoustic speed of gas samples.

**Table 1:** Acoustic properties of different gas samples:

Gas sample	Density (kg/m^3^)	Acoustic speed (m/s)	Reference
SO_2_	2.279	201	^35^
CO_2_	1.8393	267	^35^
C_3_H_8_	1.8102	249	^35^
Ar	1.661	319	^35^
O_2_	1.314	326	^35^
Air	1.2047	343	^35^
N_2_	1.165	349	^24^
NH_3_	0.7069	430	^35^
CH_4_	0.659	445	^35^

**Figure 2:**
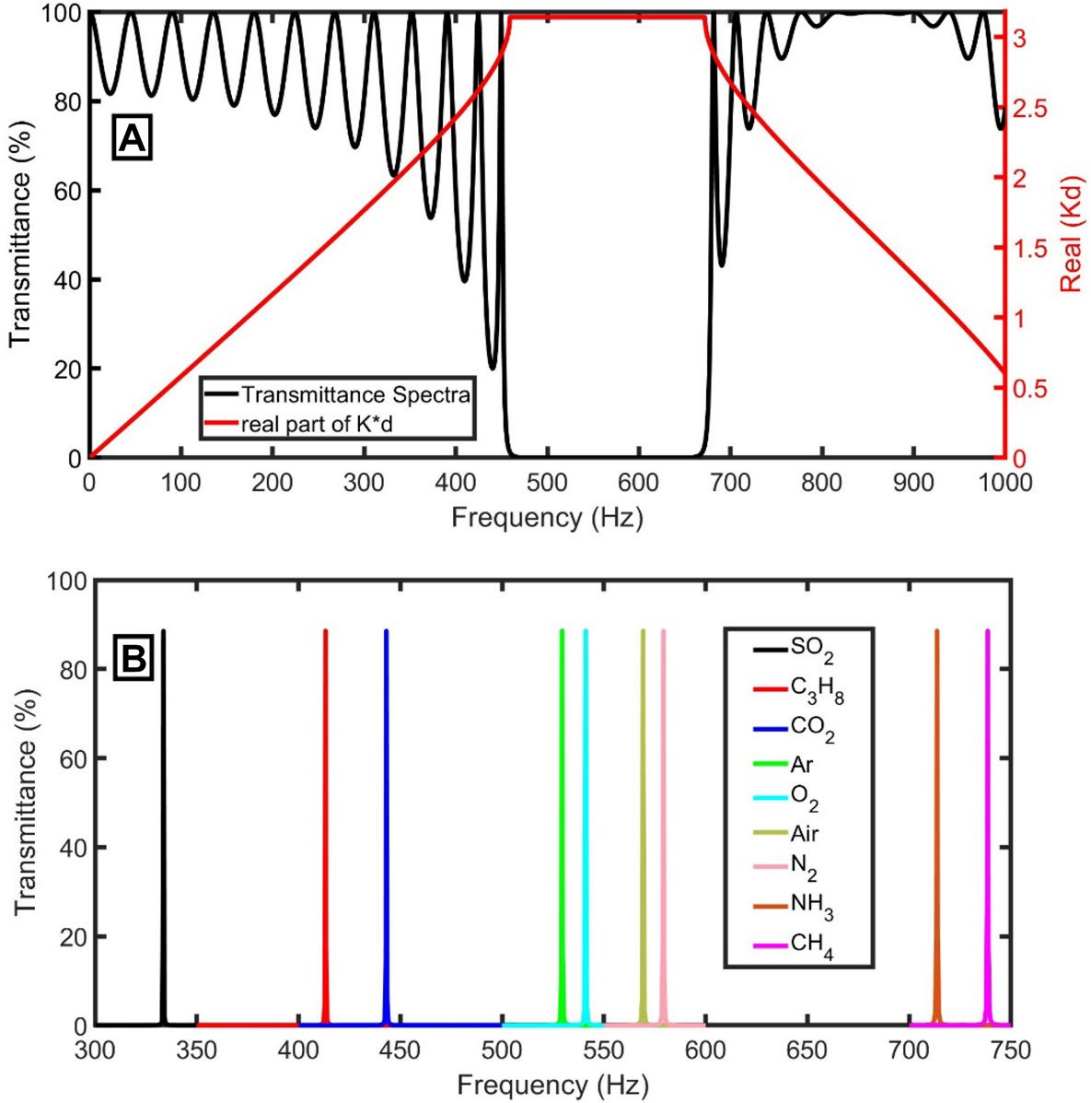
(**A**) The transmittance spectra of the proposed gas sensor without defect (left axis) and the band structure of the real part of the Bloch wavenumber (right axis) using air samples, and (**B**) the defect peaks of different gas samples by using defect.

For a perfect PnC *(M*_*1*_*M*_*2*_*)*^*N*^* (M*_*1*_*M*_*2*_*)*^*N*^ without the defect tube, an ideal PnBG is extended from 460 to 670 Hz, as evident in Fig. 2A. Within this range, the propagation of acoustic waves is forbidden. Besides the plotting of the transmittance spectra of acoustic waves, the dispersion relation is studied according to Bloch’s theorem as the following^19^:

7 $$\mathrm{cos}\left(Kd\right)=\mathrm{cos}\left(k{d}_{1}\right)\mathrm{cos}\left(k{d}_{2}\right)- \frac{1}{2}\left[m+\frac{1}{m}\right]\mathrm{sin}\left(k{d}_{1}\right)\mathrm{sin}\left(k{d}_{2}\right)$$

where $d$ is the thickness of the elementary unit cell, $K$ is the Bloch wave propagation vector, *k* is the wave vector, and $m=\frac{{S}_{2}}{{S}_{1}}$. The real part of $K$ is used to study the change in the propagating phase of waves in a pass band. Figure 2A shows that the PnBG of the transmittance spectra coincides with the band structure of the real part of the Bloch wavenumber.

As straightforward in Fig. 2B, due to the existence of a defect tube with a cross-section of 0.15 m^2^ and thickness of 0.15 m, a sharp resonant peak to the change in the nature of the sample of gas inside the tube of the proposed sensor appeared inside the PnBG due to the localization of acoustic waves in the defect layer. With the change of the gas sample from SO_2_ to C_3_H_8_, CO_2_, Ar, O_2_, Air, N_2_, NH_3_, and CH_4_, the PnBG extended from 265 to 392 Hz (SO_2_ gas sample), from 326 to 486 Hz (C_3_H_8_ gas sample), from 350 to 517 Hz (CO_2_ gas sample), from 418 to 621 Hz (Ar gas sample), from 430 to 635 Hz (O_2_ gas sample), from 449 to 671 Hz (Air sample), from 460 to 681 Hz (N_2_ gas sample), from 563 to 842 Hz (NH_3_ gas sample), and from 582 to 873 Hz (CH_4_ gas sample), respectively. Besides, the defect mode is generated at 333.6 Hz, 413.2 Hz, 443.1 Hz, 529.4 Hz, 541.1 Hz, 569.3 Hz, 579.2 Hz, 713.7 Hz, and 738.6 Hz, respectively.

Although the peak shift depends on both the mass density and acoustic speed of each sample, the sensitivity will be calculated as a function of mass density during the optimization process^24,35,36^. The sensitivity (*S*) of the investigated gas PnC sensor is represented as the ratio between the peak shift for each gas sample and the change in mass density ($$\Delta {\rho }_{gas}$$). The figure of merit (*FoM*) is the ratio between S and the bandwidth (FWHM) of the resonant mode. The quality factor (Q-factor) is the ratio between the position of the resonant mode ($${f}_{R})$$ and the bandwidth. The limit of detection (*LoD*) is the lowest mass density change that can be reliably detected. *S*, *FoM*, *Q-factor*, and *LoD* can be calculated as the following to evaluate the performance of the selected conditions:

8 $$S=\frac{\Delta {f}_{R}}{\Delta {\rho }_{gas}}=\left|\frac{{f}_{air}-{f}_{CH4}}{{\rho }_{air}-{\rho }_{CH4}}\right|,$$

9 $$FoM=\frac{S}{FWHM} ,$$

10 $$\begin{array}{l}Q=\frac{{f}_{R}}{FWHM}\end{array} ,$$

11 $$LoD=\frac{{f}_{R}}{20 S Q} .$$

In the following optimization process, the change between the resonant peak of air and pure CH_4_ samples will be used for sensitivity calculations. The FWHM will be calculated as an indicator for the resonant peak of air samples.

**Figure 3:**
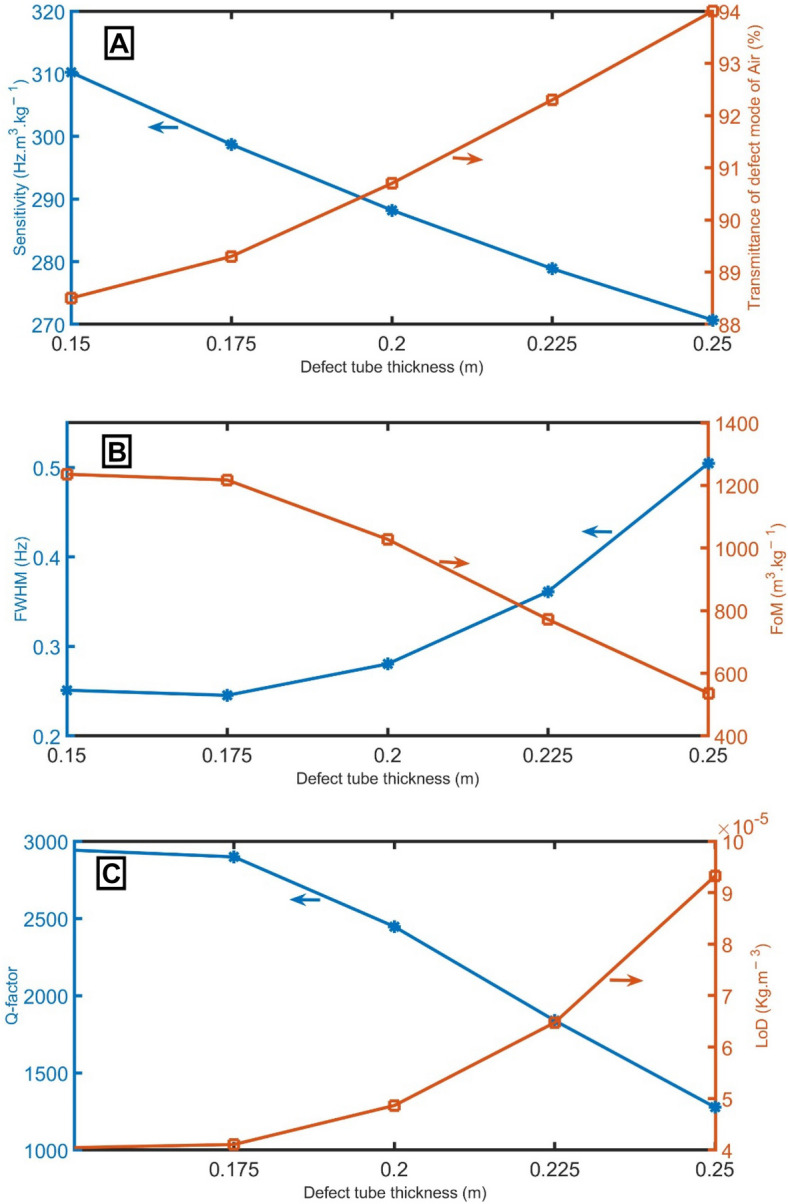
(**A**) sensitivity between air and pure CH_4_ samples and transmittance of the resonant mode of air samples, (**B**) *FWHM* of the resonant mode of air samples and *FoM*, and (**C**) *Q-factor* and *LoD* of the proposed gas sensor at different values of defect tube thickness.

Figure 3 shows the sensitivity, transmittance of the resonant mode of air samples, *FWHM* of the resonant mode of air samples, *FoM*, *Q-factor*, and *LoD* of the proposed sensor at different defect tube thickness considerations from 0.15 to 0.25 m. Outside the scope of this range of defect tube thickness, the resonant peaks overlap (Supplementary Data: Sup. 1). At a frequency range of 0 Hz < *f* < 1000 Hz, the PnBG and the resonant peak are shifted to lower frequencies (red-shift), and the sensitivity is decreased from 310 to 271  Hz m^3^ kg^−1^ with the increase of the thickness of the defect tube, as evident in Fig. 3A. The resonant peaks for which the transmittance varies between 89 and 94% are shifted to lower frequencies^37^ from 738.56 Hz (at *d*_*D*_ = 0.15 m), 711.13 Hz (at *d*_*D*_ = 0.175 m), 686.16 Hz (at *d*_*D*_ = 0.2 m), 663.93 Hz (at *d*_*D*_ = 0.225 m), and 644.47 Hz (at *d*_*D*_ = 0.25 m). The transmittance of the sample resonant peaks slightly increases with the defect tube thickness increase due to the acoustic wave confinement inside the sensor tube. As explicit in Fig. 3B, a change in the thickness of the defect tube leads to a change in the *FWHM* of the transmitted peaks. The *FWHM* increases from 0.25 to 0.50 Hz with the increase of the thickness of the defect tube from 0.15 to 0.25 m. As the *FoM*, *Q-factor*, and *LoD* are dependent parameters; their behaviors can be predicted according to Eqs. (9, 10, 11), as apparent in Fig. 3A–C. Therefore, a thickness of 0.15 m will be the optimum value.

**Figure 4:**
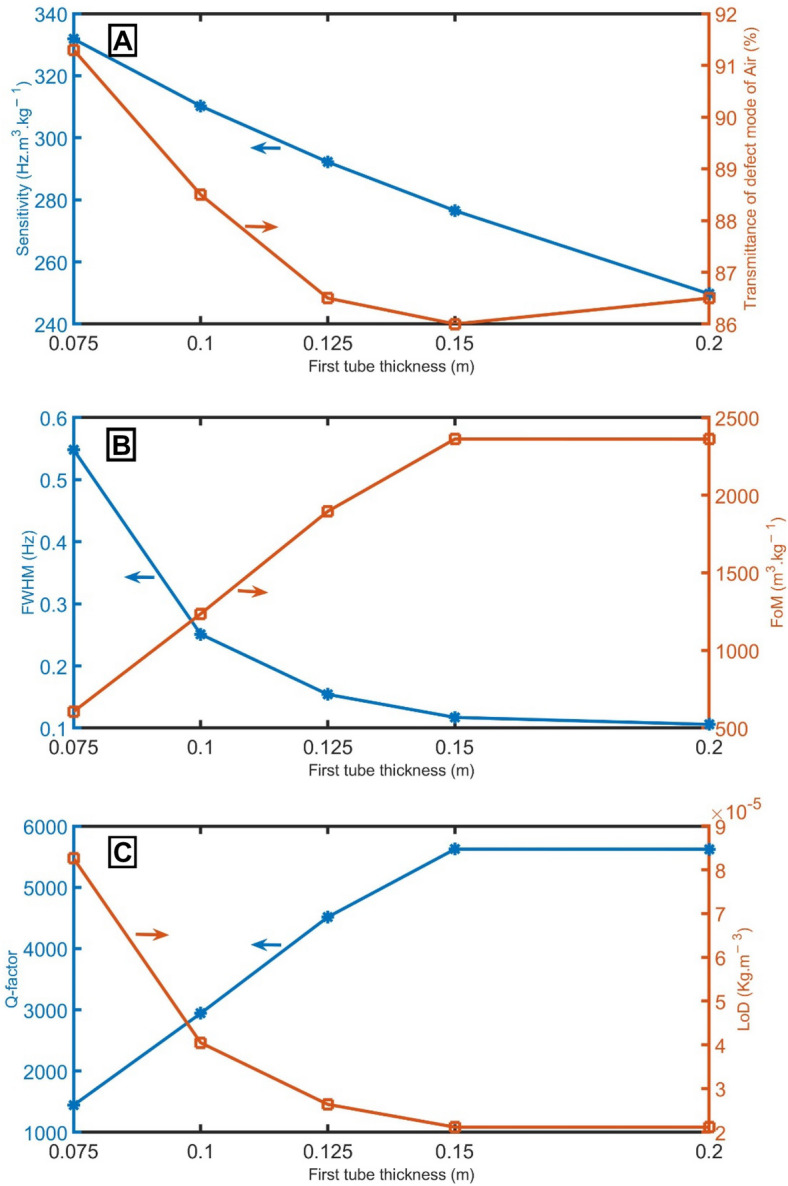
(**A**) sensitivity between air and pure CH_4_ samples and transmittance of the resonant mode of air samples, (**B**) *FWHM* of the resonant mode of air samples and *FoM*, and (**C**) *Q-factor* and *LoD* of the proposed gas sensor at different values of first tube thickness of the unit cell.

Figure 4A represents the decrease in sensitivity of the proposed detector with the increase of the first tube thickness of the unit cell. Figure 4A shows the slight change in the transmittance of the resonant mode of air samples at different values of the first tube thickness of the unit cell. When the first tube thickness of the unit cell changes from 0.075 to 0.2 m, the *FWHM* decreases from 0.55 to 0.11 Hz, as evident in Fig. 4B. As a result of the behavior of sensitivity and *FWHM*, *FoM* and *Q-factor* record the highest value at a thickness of 0.15 m, then seems to be constant, as apparent in Fig. 4B,C. On the other hand, the *LoD* decreases to the minimum value at a thickness of 0.15 m and seems to become constant at higher thicknesses, as evident in Fig. 4C. Therefore, the thickness of 0.075 m is better because the proposed sensor recorded the most heightened sensitivity.

**Figure 5:**
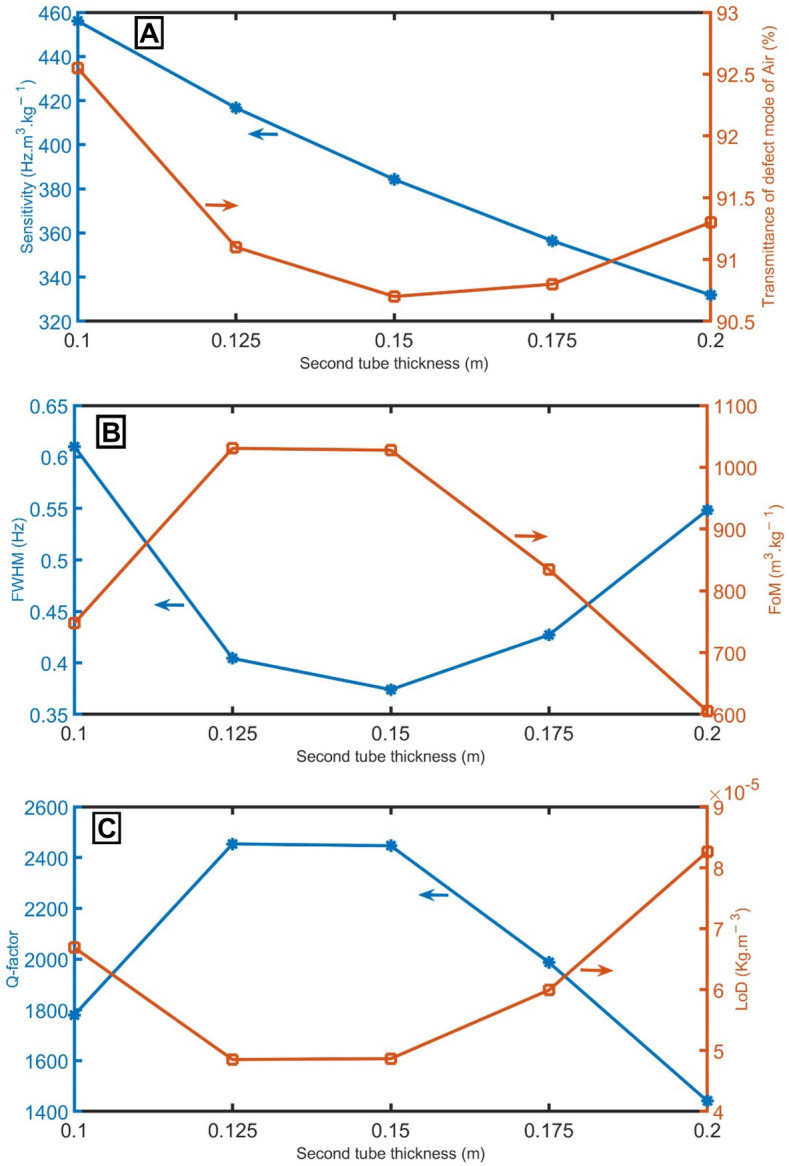
(**A**) sensitivity between air and pure CH_4_ samples and transmittance of the resonant mode of air samples, (**B**) *FWHM* of the resonant mode of air samples and *FoM*, and (**C**) *Q-factor* and *LoD* of the proposed gas sensor at different values of second tube thickness of the unit cell.

Figure 5 shows the performance of the proposed sensor at different values of the second tube thickness considerations from 0.1 to 0.2 m. At a frequency range of 0 Hz < f < 1500 Hz, the sensitivity is decreased from 456 to 332 Hz m^3^ kg^−1^ with the increase of the thickness of the second tube, as clear in Fig. 5A. The position of resonant peaks was found at 1085.86 Hz (at d_D_ = 0.1 m), 992.01 Hz (at *d*_*D*_ = 0.125 m), 914.89 Hz (at *d*_*D*_ = 0.15 m), 848.57 Hz (at *d*_*D*_ = 0.175 m), and 790.07 Hz (at *d*_*D*_ = 0.2 m). The transmittance of the resonant peaks at these thicknesses slightly varies between 90 and 93%, as clear in Fig. 5A. As straightforward in Fig. 5B, a change in the thickness of the second tube leads to a change in the *FWHM* of the transmitted dip. The *FWHM* decreases from 0.61 to 0.37 Hz with the increase of the thickness of the second tube from 0.1 to 0.15 m. After that, the *FWHM* gradually increases to 0.55 Hz. As the *FoM*, *Q-factor*, and *LoD* are dependent parameters on sensitivity and *FWHM* according to Eqs. (9, 10, 11), their behaviors depend on sensitivity and *FWHM*, as clear in Fig. 3B,C. As the highest sensitivity is at the thickness of 0.1 nm, this thickness of the second tube will be optimum.

**Figure 6:**
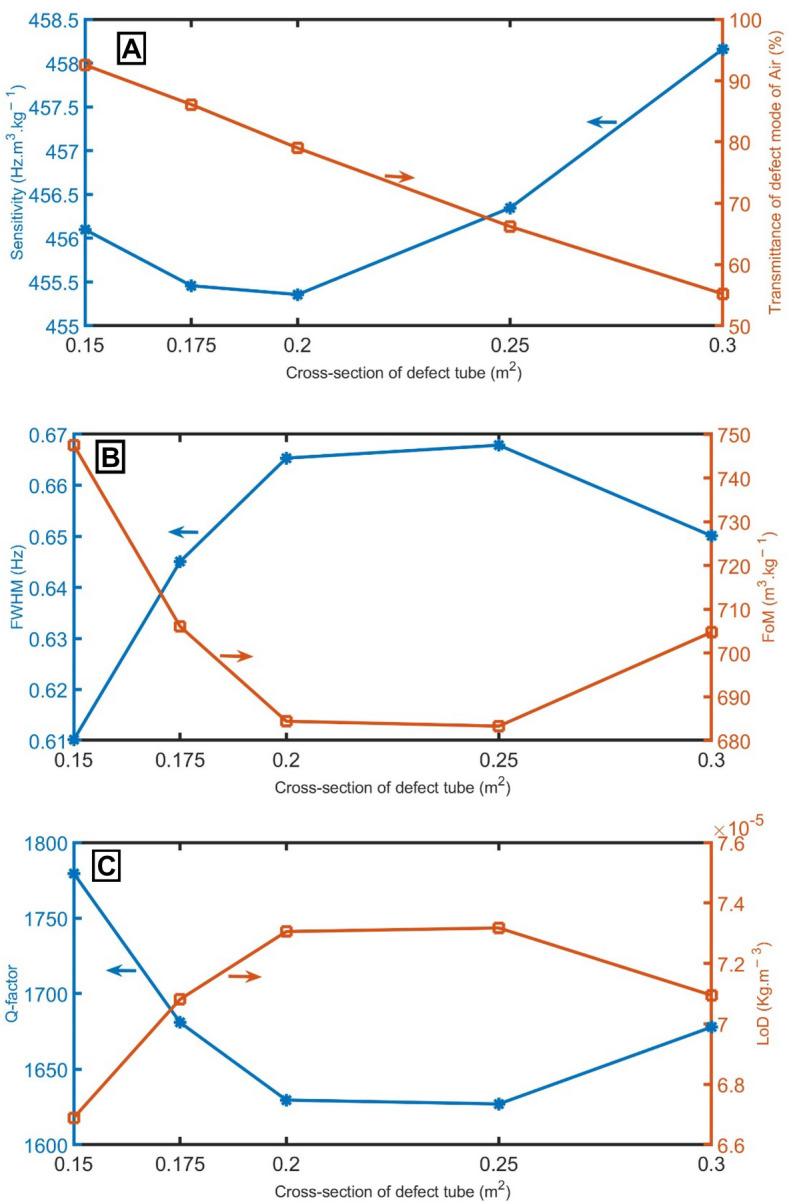
(**A**) sensitivity between air and pure CH_4_ samples and transmittance of the resonant mode of air samples, (**B**) *FWHM* of the resonant mode of air samples and *FoM*, and (**C**) *Q-factor* and *LoD* of the proposed gas sensor at different values of cross-sections of the defect tube.

Figure 6A represents the slight change in sensitivity of the proposed detector with the change in the cross-sections of the defect tube. Figure 6A shows that the transmittance of the resonant mode of air samples drops quickly with the increase of the cross-sections of the defect tube. The transmittance decreases from 92.5 to 55.2% with the rise of the cross-sections of the defect tube from 0.15 to 0.3 m^2^. When the cross-section of the defect tube changes from 0.15 to 0.25 m^2^, the *FWHM* increases from 0.61 to 0.67 Hz, as clear in Fig. 6B. Then, the *FWHM* decreases to 0.65 Hz at a cross-section of 0.3 m^2^. As a result of the behavior of sensitivity and *FWHM*, *FoM* and *Q-factor* record the lowest value of 683 m^3^/kg and 1627 at a thickness of 0.25 m, then decreases to 705 m^3^/kg and 1678 at cross-section of 0.3 m, as clear in Fig. 6B,C respectively. On the other hand, the *LoD* increases to a maximum value at a cross-section of 0.25 m^2^ and seems constant at higher cross-sections, as clear in Fig. 4C. The cross-section of 0.15 m^2^ is the best because the proposed sensor recorded the lowest *FWHM*, highest *FoM*, highest transmittance, highest *Q-factor*, and lowest *LoD*.

**Figure 7:**
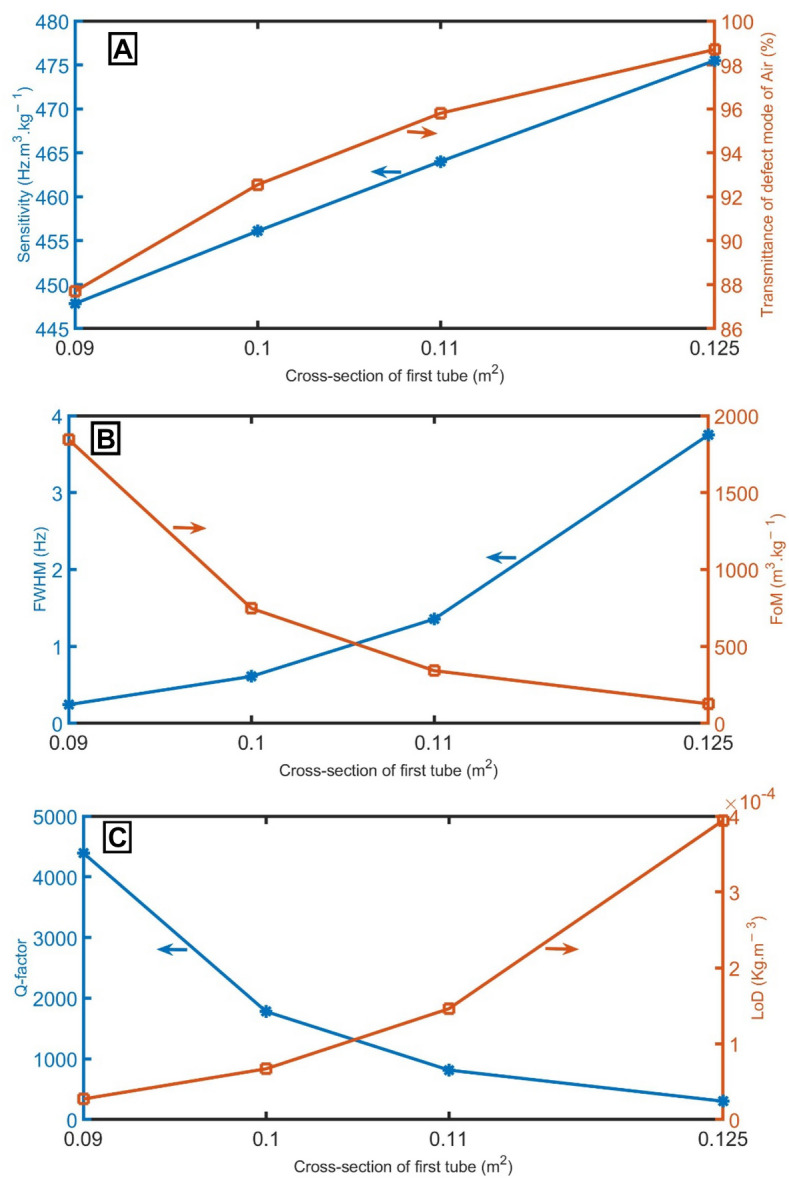
(**A**) sensitivity between air and pure CH_4_ samples and transmittance of the resonant mode of air samples, (**B**) *FWHM* of the resonant mode of air samples and *FoM*, and (**C**) *Q-factor* and *LoD* of the proposed gas sensor at different cross-sections of the first tube of the unit cell.

Figure 7A shows the sensitivity when the cross-section of the first tube of the unit cell is *S*_*1*_ = 0.09 m^2^, *S*_*1*_ = 0.1 m^2^, *S*_*1*_ = 0.11 m^2^, and *S*_*1*_ = 0.125 m^2^. As the cross-section of the first tube gradually increases, it can be seen that the resonant peak undergoes a blue shift to higher frequencies, and the sensitivity increases gradually from 448 to 476 Hz m^3^ kg^−1^. This behavior is because the cross-section of the first tube of the unit cell is large, the impedance of the proposed structure to the acoustic wave decreases, and the PnBG containing the defect peak is blue-shifted to higher frequencies, resulting in an increase in sensitivity. Above the cross-section of 0.125 m^2^, the resonant peak goes out from the PnBG. Besides, the transmittance of the peaks increases from 87.7 to 97.7% with the increase of the cross-section of the first tube of the unit cell from 0.09 to 0.125 m^2^, as clear in Fig. 7A. As regards the impact of the increase of the cross-section of the first tube of the unit cell on the *FWHM*, the *FWHM* increases from 0.24 to 3.75 Hz with the increase of the cross-section of the first tube of the unit cell from 0.09 to 0.125 m^2^, as clear in Fig. 7B. In Fig. 7B,C, the *FoM* and *Q-factor* decrease with the increase of the cross-section of the first tube. For *LoD*, it increases with the increase of the cross-section of the first tube, as clear in Fig. 7C. The cross-section of 0.125 m^2^ is optimum.

**Figure 8:**
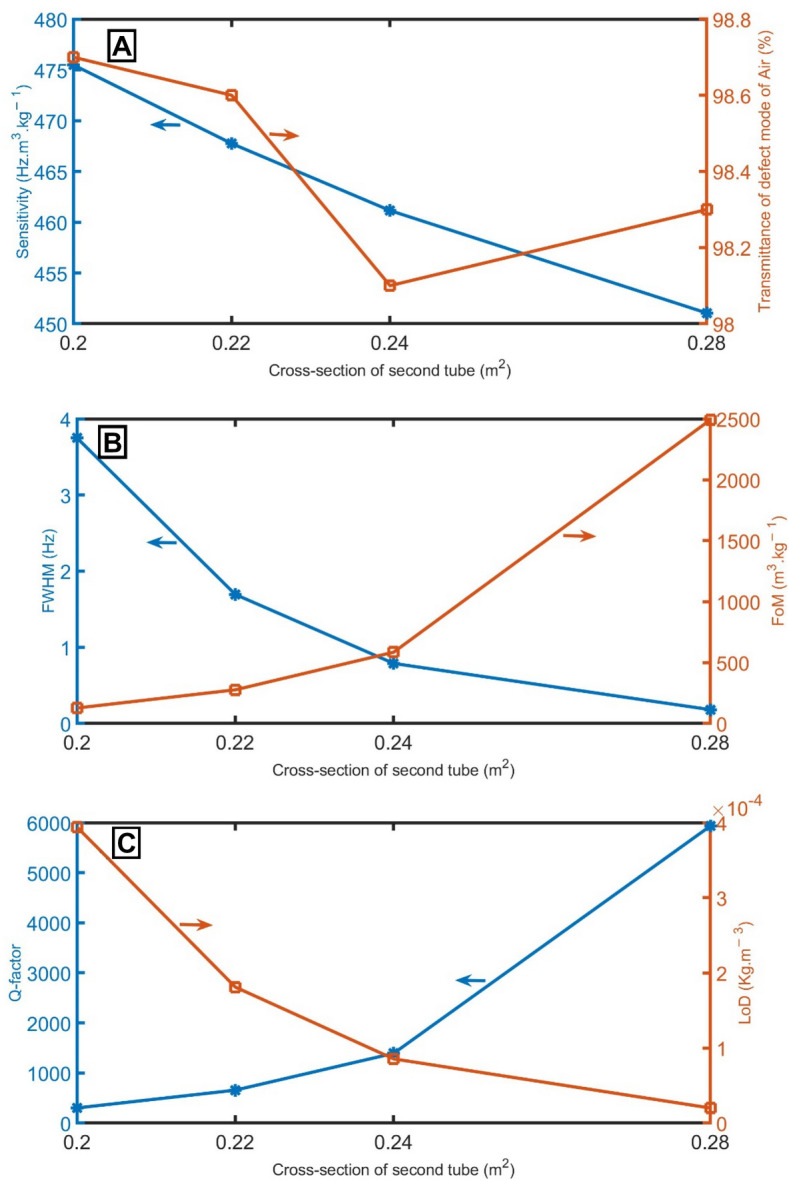
(**A**) sensitivity between air and pure CH_4_ samples and transmittance of the resonant mode of air samples, (**B**) *FWHM* of the resonant mode of air samples and *FoM*, and (**C**) *Q-factor* and *LoD* of the proposed gas sensor at different cross-sections of the second tube of the unit cell.

Similarly, Fig. 8A gives the impact of the cross-section of the second tube of the unit cell on sensitivity at *S*_*2*_ = 0.2 m^2^, *S*_*2*_ = 0.22 m^2^, *S*_*2*_ = 0.24 m^2^, and *S*_*2*_ = 0.28 m^2^. As demonstrated, the sensitivity is slightly decreased from 475.5 to 451.1 Hz m^3^ kg^−1^ with the increase of the cross-section of the second tube. Figure 8A gives the relation between the transmittance of the resonant peaks and the cross-section of the second tube. Clearly, the transmittance is slightly affected by the change in the cross-section of the second tube from 0.20 to 0.28 m^2^. In contrast, the *FWHM* strongly decreases from 3.75 to 0.18 Hz with increasing the cross-section of the second tube from 0.20 to 0.28 m^2^, as clear in Fig. 8B. Figure 8B gives the *FoM* of the proposed sensor as a function of the cross-section of the second tube. The highest and lowest values of *FoM* are at *S*_*2*_ = 0.28 m^2^ and 0.20 m^2^, respectively. Similar to Fig. 8B, the *Q-factor* changes dramatically with the cross-section of the second tube from 0.20 to 0.28 m^2^, as clear in Fig. 8C. In the case of the *LoD*, with the increase of the cross-section of the second tube from 0.20 to 0.28 m^2^, the *LoD* dramatically decreases from 4 × 10^–4^ to 2 × 10^–5^ $\mathrm{kg }{\mathrm{m}}^{-3}$, as clear in Fig. 8C.

**Figure 9:**
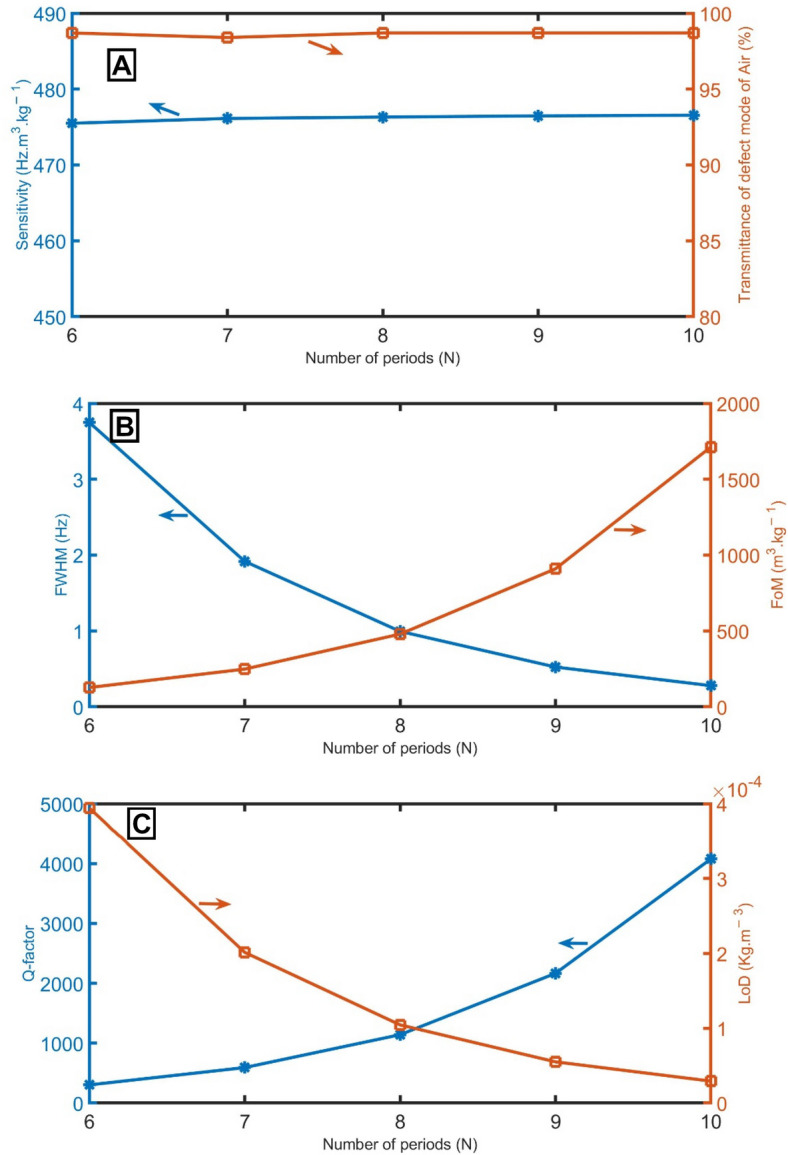
(**A**) sensitivity between air and pure CH_4_ samples and transmittance of the resonant mode of air samples, (**B**) *FWHM* of the resonant mode of air samples and *FoM*, and (**C**) *Q-factor* and *LoD* of the proposed gas sensor at different numbers of unit cells.

Figure 9A demonstrates the sensitivity when the numbers of unit cells are *N* = 6, *N* = 7, *N* = 8, *N* = 9, and *N* = 10. As the number of unit cells increases, the resonant peak undergoes a slight blue shift to higher frequencies, and the sensitivity increases modestly from 475.5 to 476.5 Hz m^3^ kg^−1^. The resonant peak for *N* smaller than 5 goes out from the PnBG. In Fig. 9A, the transmittance of the peaks seems to be constant (98.5%) with the increase in the number of unit cells. As regards the impact of the unit cell number on the *FWHM*, the *FWHM* decreases from 3.75 to 0.29 Hz with the increase of the unit cell number from *N* = 6 to *N* = 10, as clear in Fig. 9B. In Fig. 9B,C, the *FoM* and *Q-factor* increase with the increase of the unit cell number. For *LoD*, it decreases with the increase of the unit cell number, as clear in Fig. 9C.

Even though there is no contrast in the mass density of the two tubes of each unit cell (both will be filled with the same gas sample), their acoustic impedances are different due to the change in cross sections according to Eq. (4). Due to the periodicity of the acoustic impedance, the PnBG was formed as clear in Fig. 2A. By adding a defect tube with a different cross-section, the periodicity of the acoustic impedance was broken, and the acoustic waves were localization inside the defect tube, as clear in Fig. 2B. The position of the PnBG and PnC resonant mode depends on the amount of energy localized in the defect tube, which depends on the acoustic properties of the gas filling it. So, by knowing the position of the localized state position within the PnBG, an unknown gas can be detected because each gas has a distinguished localized state position.

In general, PnBG and resonant peak were red-shifted with the increase of thickness (to lower frequencies) according to the standing wave equation:

12 $$2d=\frac{n c}{f},$$

where *d* is the thickness, *n* is an integer, c is the acoustic velocity, and *f* is the frequency. According to Eq. (12), the frequency is inversely proportional to the thickness of the tubes. Besides, the sensitivity of the proposed sensor decreases with the increase of the thickness of any tube. This negative thickness behavior may be related to the acoustic path and the attenuation of the wave within the tube.

For the cross-section of the tubes, according to Eq. (4), the acoustic impedance is inversely proportional to the cross-section of tubes, and the interaction between the acoustic wave and the gas sample increases with the decrease of the cross-section. Although, we noticed that the sensitivity sometimes increased with the increase of the cross-section and other times decreased. For both the first and second tubes of the unit cell, we found that the proposed sensor recorded high sensitivity when the cross-section of the first tube has a broad cross-section and the second tube has a small cross-section. For the defect tube cross-section, the sensitivity increases with the cross-section because this increase in the defect cross-section increased the contraction between the defect layer and the next layer to it (the first layer of the unit cell). When we tried to increase or decrease the thicknesses or cross-sections from the selected ranges, the resonant peaks overlapped or went out from the PnBG.

**Figure 10:**
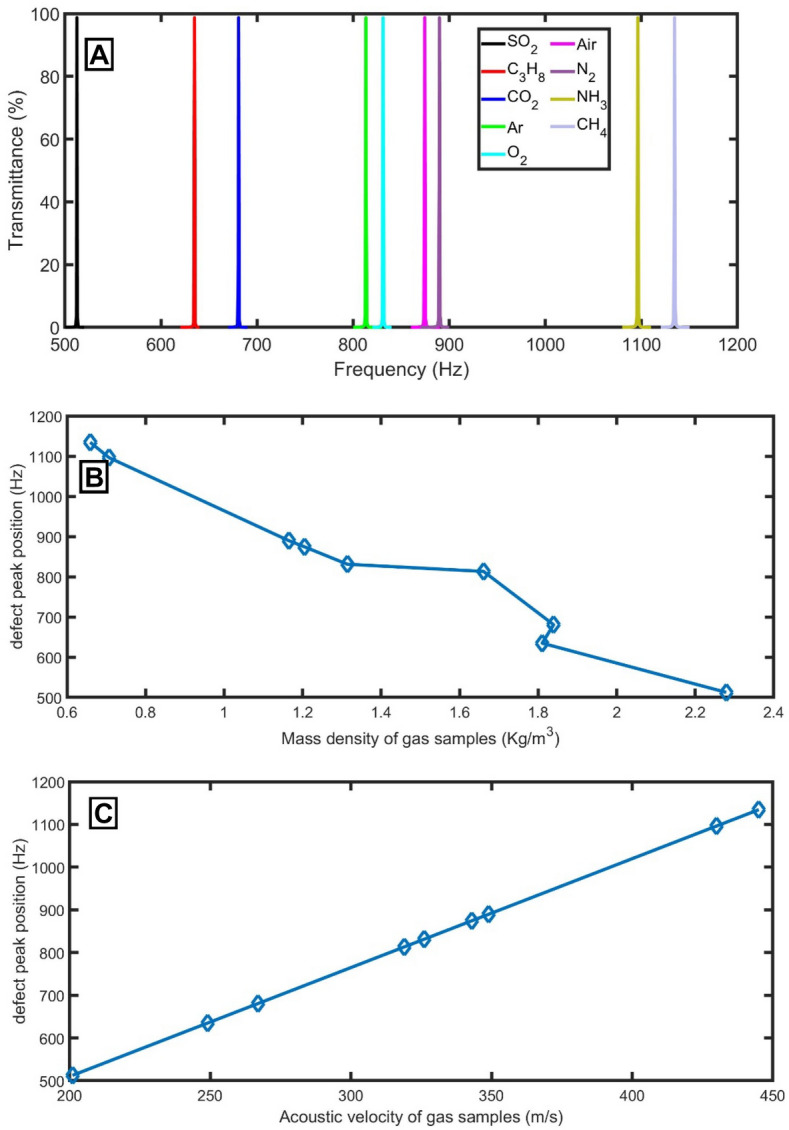
(**A**) the transmittance of different gas samples at the selected conditions, (**B**) the defect peak position versus the mass density for each gas sample, and (**C**) the defect peak position versus the acoustic velocity for each gas sample.

Figure 10A shows the transmittance of different gas samples at the selected conditions. As clear in Fig. 10B,C, the acoustic velocity is a significant parameter rather than the mass density variations of the gas samples on the position of the resonant peaks due to its significant impact on the propagation of the acoustic wave (Eq. 1). On the other hand, the change in the mass density of the gas samples is tiny, as evident in Table 1. As a result, the acoustic velocity is an excellent choice to evaluate the performance of the proposed sensor because the mass density does not have a linear relation with the position of the resonant peaks as occurred in the case of the acoustic velocity. So, we will re-calculate the sensitivity of the sensor at the optimum conditions as the ratio between the resonant peak shift and the change in the acoustic velocity as the following:

13 $$S=\frac{\Delta {f}_{R}}{\Delta {C}_{gas}}=\left|\frac{{f}_{air}-{f}_{CH4}}{{C}_{air}-{C}_{CH4}}\right|,$$

The linear relation between the position of the resonant peak and the acoustic velocity of the gas sample can be described as the following:

14 $${f}_{R}=2.5495 c - 0.0096,\quad {R}^{2}=1$$

According to Eq. (14), the sensitivity of the proposed sensor is constant (2.5495 Hz s m^−1^) for all gas samples.

### Concentration of CO_2_ in air calculations

To calculate the mass density of a mixture of air and CO_2_ as a function of the concentration of CO_2_, the following equation will be used^38^:

15 $${\rho }_{mix}= {{\rho }_{CO2} {\alpha }_{CO2}+\rho }_{air} (1-{\alpha }_{CO2}),$$

where ${\rho }_{mix}$ is the density of the mixture, ${\rho }_{air}$ is the density of the air, ${\rho }_{CO2}$ is the density of the CO_2_, ${\alpha }_{air}$ is the volume fraction of air and ${\alpha }_{CO2}$ is the volume fraction of CO_2_. The following relation calculates the longitudinal sound speed of the mixture of air and CO_2_ as a function of the temperature (at *T* = 20 K), the ratio of CO_2_ in the air (${\alpha }_{CO2}$), the molecular weights of air ($m_{air}$) and CO_2_ ($m_{CO2}$), and specific heat capacity at constant pressure ($C_{p1}$ and $C_{p2}$) and volume ($C_{v1}$ and $C_{v2}$)^38^:

16 $${C}_{mix}=\sqrt{\left(\frac{R}{{m}_{air}\left(1-{\alpha }_{CO2}\right)+{m}_{CO2} {\alpha }_{CO2}}\right)\left(\frac{{C}_{p1}\left(1-{\alpha }_{CO2}\right)+{C}_{p2}{\alpha }_{CO2}}{{C}_{v1}\left(1-{\alpha }_{CO2}\right)+{C}_{v2}{\alpha }_{CO2}}\right)T},$$

where *R* is the gas constant 8.314 J/mol-K. By changing the ratio of CO_2_ in the air from 0 to 20%, 40%, 60%, 80%, and 100%, the longitudinal sound speed of the mixture changes from 343 m/s to 325.1 m/s, 307.6 m/s, 290.3 m/s, 279.9 m/s, and 273.4 m/s, respectively.

**Figure 11:**
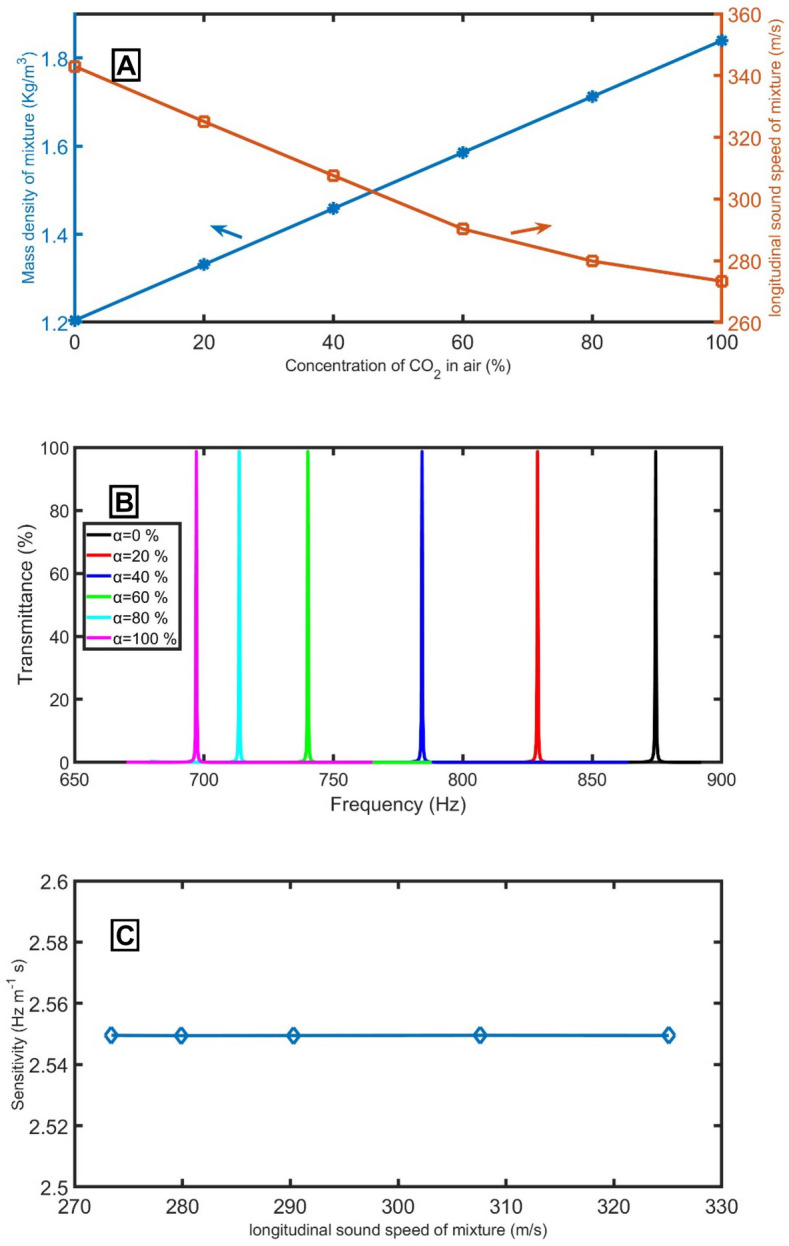
(**A**) the mass density of the mixture and longitudinal sound speed of the mixture versus the concentration of CO_2_, (**B**) the transmittance of different ratios of CO_2_ in the air at the optimum conditions, and (**C**) the sensitivity versus the acoustic velocity at different concentrations.

Figure 11A clears the mixture's mass density and the mixture's longitudinal sound speed versus the concentration of CO_2_ in the air. With increasing the ratio of CO_2_ in the air from 0 to 20%, 40%, 60%, 80%, and 100%, the position of the resonant peak is shifted from 874.5 Hz to 828.8 Hz, 784.2 Hz, 740.1 Hz, 713.6 Hz, and 697.0 Hz, as clear in Fig. 11B. the proposed sensor recorded high stability (fixed sensitivity) for all concentrations, as clear in Fig. 11C.

The main advantage of the proposed sensor is:

1. The structure is very simple and low-cost to be fabricated compared to many other models because it does not need a complicated process to deposit a multi-tube of different mechanical properties’ materials. It is simple alternating tubes with different cross-sections in periodic sequences.
2. The proposed sensor does not need a recovery time.
3. The suggested structure recorded very high linearity between the change in the resonant peak and the acoustic velocity of the sample.

## Conclusion

In summary, the proposed PnC gas sensor depends on the contrast in the acoustic velocities between gas samples with tube sequence of *(M*_*1*_*M*_*2*_*)*^*N*^* M*_*D*_* (M*_*1*_*M*_*2*_*)*^*N*^. The transmittance spectra of the interaction between the incident acoustic wave and the proposed PnC structure were studied using the TMM. All geometrical parameters are optimized to achieve the highest performance. The proposed model can monitor many samples of gases depending on the physio-chemical variations in the structure due to the interaction between the gas sample and acoustic wave inside the system. Our sensor recorded sensitivity, *Q-factor*, and *FoM* of 2.5495 Hz s m^−1^, 4077, and 9.16 s m^−1^. The main merits of the proposed sensor are the simplicity of fabrication and linearity.

## Supplementary Information

Supplementary Figure 1.

## Data Availability

All data generated or analysed during this study are included in this published article [and its supplementary information file]. The MATLAB code that support the findings of this study should be addressed to Zaky A. Zaky.
