# Supplementary material for: Simulation study of gas sensor using periodic phononic crystal tubes to detect hazardous greenhouse gases
Source: Sci Rep. 2022 Dec 13;12:21553. doi: 10.1038/s41598-022-26079-0 (PMC9747703; doi:10.1038/s41598-022-26079-0)
Supplement: Supplementary file 1 — Supplementary Figure 1. [file 41598_2022_26079_MOESM1_ESM.docx]

Simulation study of gas sensor using periodic phononic crystal tubes to detect hazardous greenhouse gases

Zaky A. Zaky^1,^*, Sagr Alamri ^2^, Ensjam I. Zohny^1^, Arafa H. Aly^1^

^1^ TH-PPM Group, Physics Department, Faculty of Science, Beni-Suef University, Egypt, 62521

^2^ Department of Mechanical Engineering, College of Engineering, King Khalid University, Abha 61421, Saudi Arabia

*Corresponding author. zaky.a.zaky@science.bsu.edu.eg (Zaky A. Zaky)

In Sup. 1(A, B, C, D, E, F, G, H, and I), for SO_2_, there are two defect peaks (peak 1 and peak 2). by changing the gas sample, the first defect peak is shifted to high frequencies. The peaks of NH_3_ (peak 3) and CH_4_ (peak 4) exceed the second peak of the SO_2_ sample. So, SO_2_ has two peaks inside the frequency range of the study. This is not good for sensing applications.


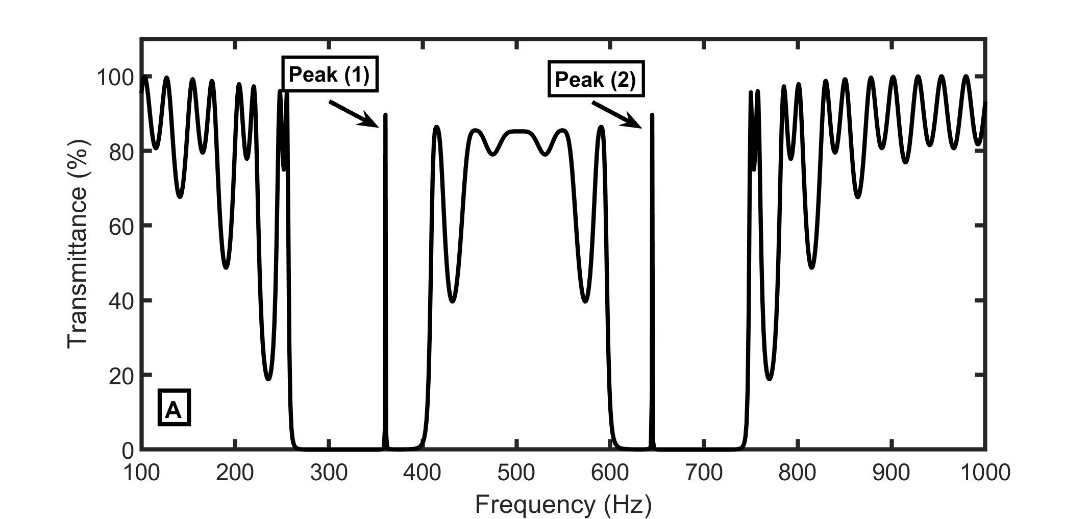


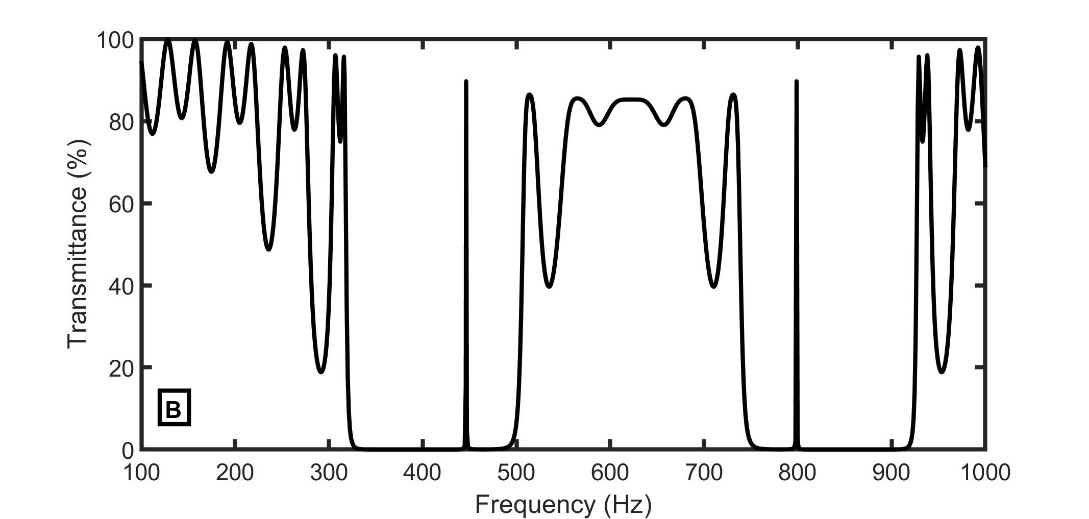

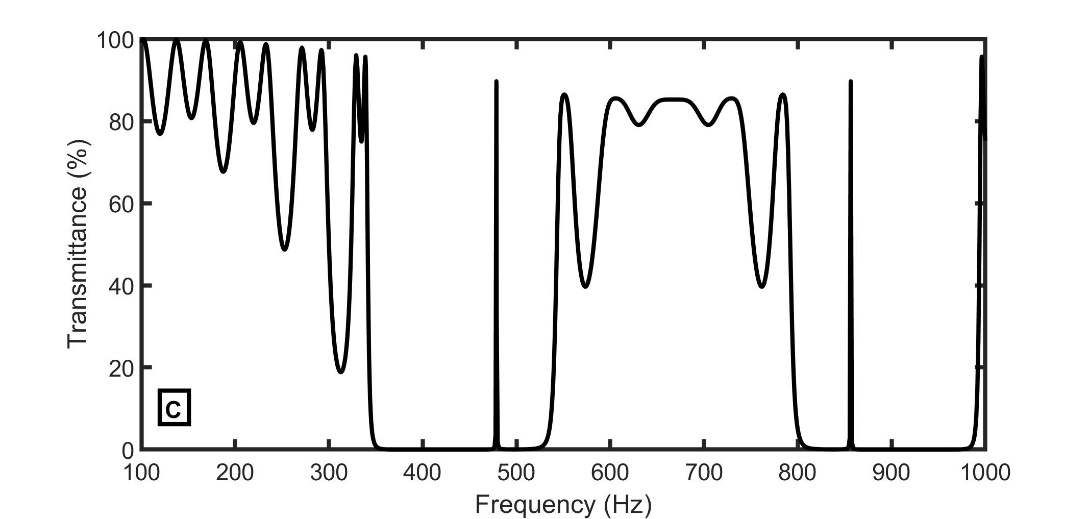

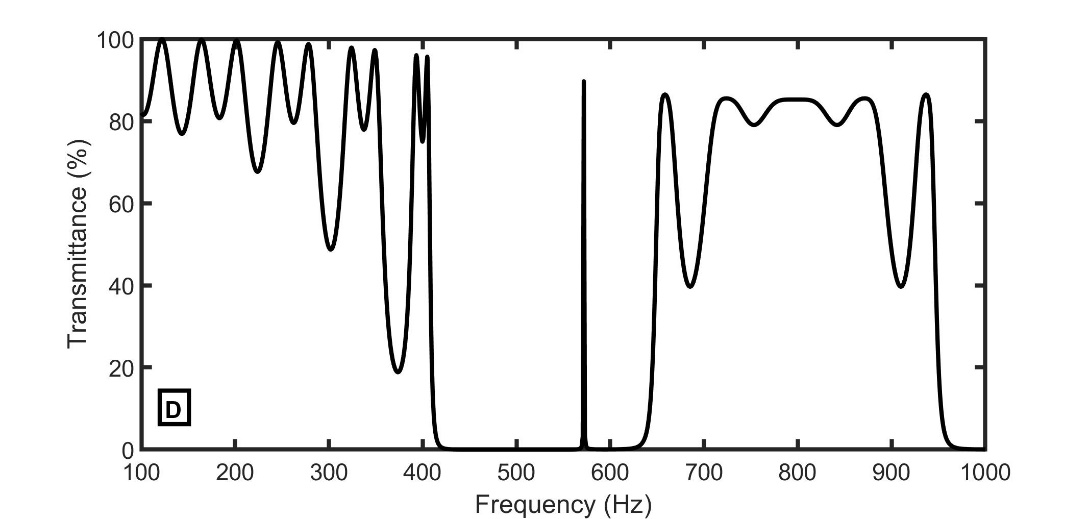

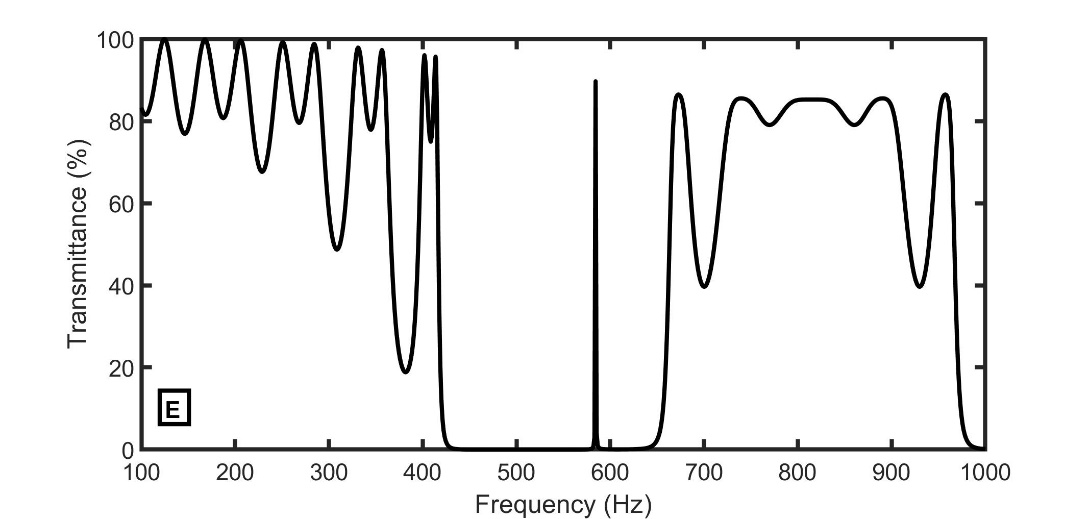

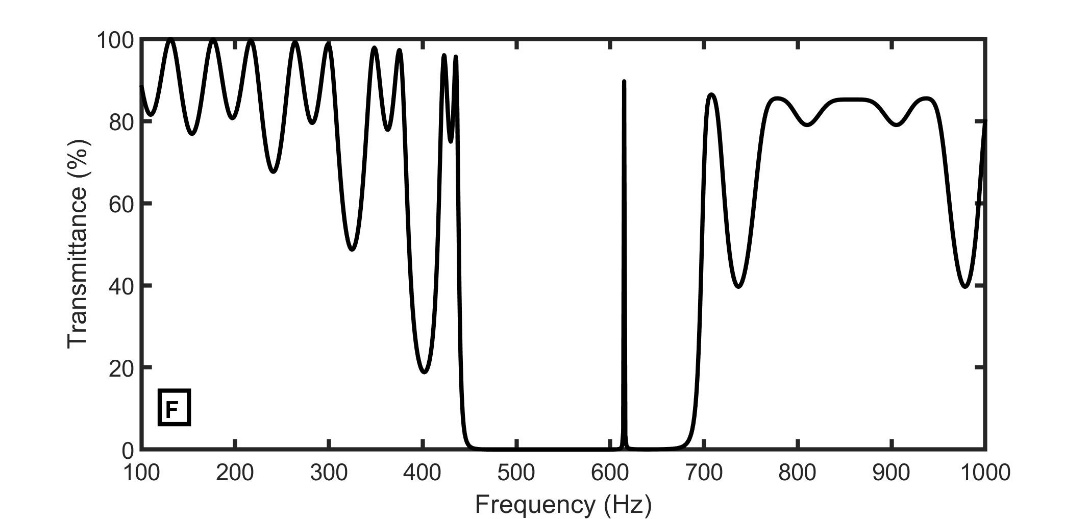

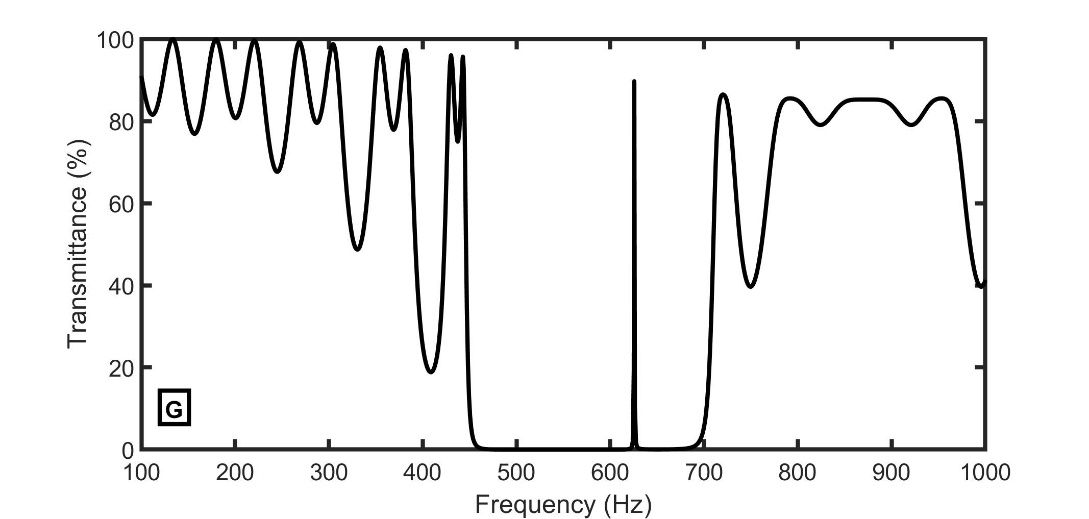

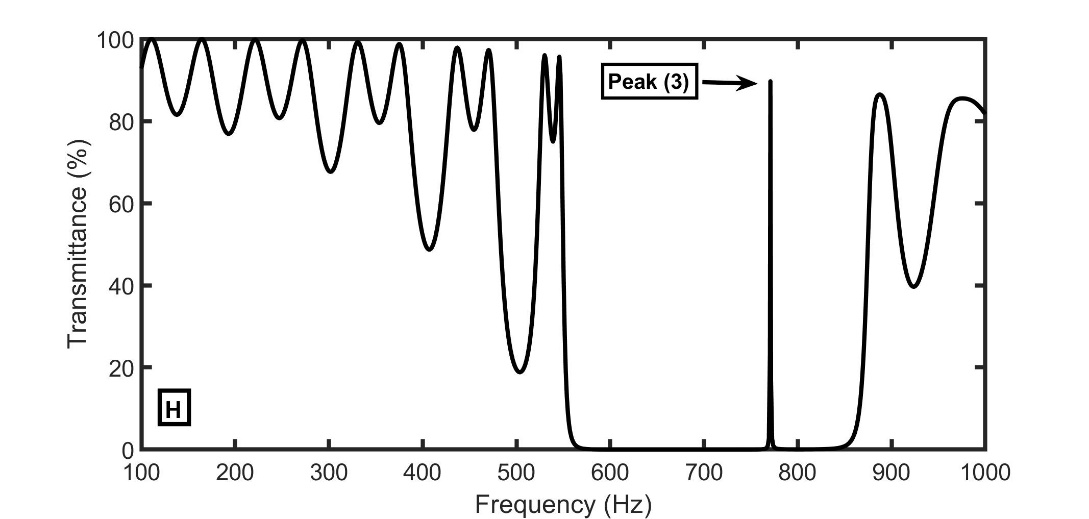

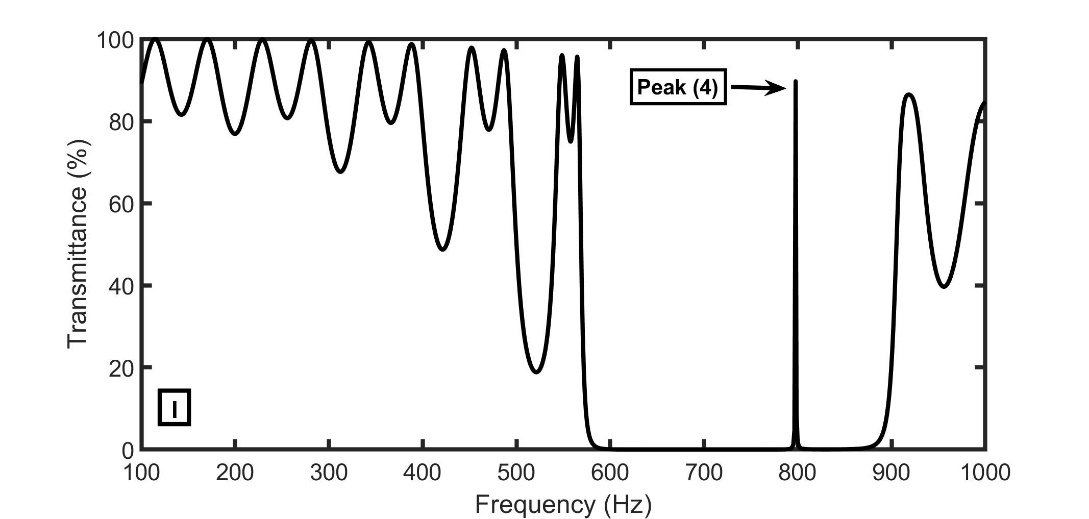


Sup. 1: The transmittance spectra of the proposed gas sensor for different gas samples (A) SO_2_, (B) CO_2_, (C) C_3_H_8_, (D) Ar, (E) O_2_, (F) Air, (G) N_2_, (H) NH_3_, and (I) CH_4_ at d_D_=0.1 mm.
